# 基因治疗在血友病B领域的研究进展

**DOI:** 10.3760/cma.j.cn121090-20250122-00041

**Published:** 2025-06

**Authors:** 铁楠 朱

**Affiliations:** 中国医学科学院 北京协和医学院 北京协和医院血液内科，北京 100730 Department of Hematology, Peking Union Medical College Hospital, Chinese Academy of Medical Sciences & Peking Union Medical College, Beijing 100730, China

## Abstract

血友病B（Hemophilia B, HB）是一种X染色体连锁隐性遗传病，因编码凝血因子Ⅸ（FⅨ）的基因突变引起凝血因子Ⅸ缺乏所致。替代治疗为目前的标准治疗措施，但面临着需终身用药、频繁静脉穿刺、输注后FⅨ活性水平存在波峰波谷影响疗效以及可能产生抑制物等诸多挑战。基因治疗是指通过基因替代或编辑技术，恢复改变功能细胞的生物学特性，以达到治疗目的的医学手段。随着多个临床试验所展现的良好疗效和安全性，针对HB的部分基因治疗产品已成功或即将上市，HB的临床治疗格局面临重要变化。本文梳理了这一领域的主要进展，并对一些重要问题加以讨论，为相关研究和实践提供参考。

## Abstract (en)

Hemophilia B（HB）is an X-chromosome-linked recessive disorder caused by mutations in the gene encoding coagulation factor Ⅸ（FⅨ）, resulting in coagulation factor Ⅸ deficiency. However, factor replacement treatment, as the standard of care in patients with HB at present, still faces many challenges such as the need for lifelong medication, frequent venipunctures, fluctuation of FⅨ activity levels after infusion, and potential risk for the development of inhibitors. Gene therapy（GT）is an approach to achieve therapeutic goals by modifying or manipulating the expression of target genes and altering the biological properties of living cells. As favorable efficacy and safety are demonstrated in various clinical trials, some GT products for HB have been successfully marketed, and the clinical treatment landscape of HB is facing important changes. This article summarizes the major advances in this field and discusses some noteworthy issues to provide references for relevant research and clinical practice.

血友病B（Hemophilia B, HB）是一种X染色体连锁隐性遗传性出血性疾病，因编码凝血因子Ⅸ（FⅨ）的基因突变引起凝血因子Ⅸ缺乏而致[1]。HB占所有血友病患者的15％～20％，全球男性发病率为3.8/10万，其中重型为1.1/10万。据估计中国血友病患病率在（2.73～3.09）/10万，而全国血友病登记系统于2007年至2021年间共登记有HB患者3 782例[2]–[4]。HB患者主要表现为关节、肌肉和深部组织出血，依严重程度不同，可为自发性出血或外伤后的严重出血。反复关节出血可能导致关节畸形，由此引发的功能障碍严重影响患者的生活质量。研究还显示，HB患者的疾病结局-生活质量评分比血友病A更低，提示HB的疾病负担可能更沉重[5]，但由于HB罕见，其危害易被临床低估。

目前，HB的标准治疗方法是FⅨ替代治疗，首选基因重组及血源FⅨ制剂或病毒灭活的血源性凝血酶原复合物，无上述条件时可选用新鲜冰冻血浆等[1]。通过修饰得到的长效FⅨ可以延长药物半衰期和提升FⅨ谷水平，进而提高HB患者预防治疗的疗效和依从性[6]。通过靶向天然抗凝通路以再平衡凝血功能的非因子替代产品目前亦于国外上市，尤其适用于已产生抑制物的患者。然而目前HB患者仍面临着多重治疗困境，包括FⅨ因子替代疗法需终身用药，频繁静脉穿刺导致患者生活质量严重下降、依从性差，以及替代疗法可导致抑制物发生的风险，而抑制物清除成功率低、影响治疗的安全等问题。特别是当前的治疗方式难以持续而稳定地维持血液FⅨ因子活性水平，患者的出血风险难以得到充分控制，需要更有效的治疗策略来应对。有利的是，HB作为一种单基因遗传病，患者体内FⅨ活性上升至5％即可使出血症状显著减轻，且通过检测血浆FⅨ活性水平即可评估基因表达情况，基因治疗的优势明确[7]，该疗法近年来已取得显著进展，成为可能彻底改变当前血友病治疗格局的潜在选择。本文梳理了HB基因治疗领域的关键概念和Ⅲ期试验进展，并对重要的临床问题加以讨论，为未来研究和实践提供参考。

一、HB基因治疗的机制

（一）基因治疗简介

基因治疗是指通过载体以外源基因替代体内突变基因，或采用基因编辑手段将受损或突变的基因修饰成序列正确的基因型，使患者体内长期稳定地产生特定蛋白质，达到治疗疾病的目的[8]。相关的临床试验数量近年来持续增长，至2023年3月，全球已完成、正在进行或已获批的临床试验达3 900项，中国占16.7％，排名居世界第二[9]。根据给药途径不同，基因治疗可分为两种[10]：①离体基因治疗：利用异体正常细胞或自体病变细胞，在体外通过基因导入的方式修饰和扩增后回输以治疗疾病；②在体基因治疗：利用病毒或非病毒载体直接将治疗基因递送到患者体内并治疗疾病。

以基于腺相关病毒（AAV）的人类HB基因疗法为例，将FⅨ目的基因整合到具有组织/器官靶向性的AAV载体上，经静脉给药，通过细胞的内吞作用，携带FⅨ基因的AAV载体进入肝细胞，随后进入胞核并脱去衣壳，AAV-FⅨ基因组几乎都以游离的形式存在，其表达的FⅨ蛋白最终释放入血[11]–[12]。

（二）增加FⅨ基因的表达

FⅨ的活性与其基因的编码序列直接相关。早期基于AAV载体的HB基因治疗临床试验，整合了野生型或密码子优化的FⅨ基因，由于FⅨ表达水平偏低或不能长期维持而未能满足临床需求[13]–[15]。2009年，意大利Padua大学附属医院首次在1例静脉血栓患者体内发现FⅨ R338L突变体（第338位精氨酸被亮氨酸取代），其FⅨ活性较野生型提高了8倍，而基因重组FⅨ（R338L）也保持了类似的高活性，被命名为FⅨ-Padua[16]。这一发现对HB基因治疗具有重要意义，因为高活性FⅨ-Padua不仅可以在一定程度上弥补野生型FⅨ因子活性不足的缺点，还可以减少载体用量并提升安全性，目前已成为FⅨ递送基因的主要选择。此外，近年来还报告了天然变体Shanghai（R338Q）或工程化变体ET9等其他高活性的FⅨ[17]–[18]，但仍处在早期探索阶段，未来能否用于临床仍有待验证。

（三）AAV载体的选择

用于基因治疗的理想载体，应具有低免疫原性、低致病性、高效基因转导、高器官靶向性等特点。既往作为载体的病毒包括逆转录病毒、慢病毒和腺病毒，但由于在宿主体内表达时间短暂、发生插入突变或容易诱发炎性反应等诸多缺陷[8]，上述载体难以在临床推广。AAV是一种微小病毒，由约4.7 kb的单链线状DNA基因组和直径约20～25 nm的二十面体蛋白质衣壳组成，与其他病毒载体相比，具有低整合性、低免疫原性、低致病性的特点，并具有在不同组织中的长期表达能力，成为了目前基因治疗的主要载体[19]–[20]。AAV具有多种血清型，其来源、受体及组织亲和力具有一定差异，为基因治疗的研究提供了灵活的选择。病毒衣壳是载体趋向性的主要决定因素，已经开发了多种策略对AAV衣壳进行改造，最终目标是创造具有改进靶向亲和力的新载体，提高基因转导的效率[21]。世界范围内基于AAV的基因治疗临床试验占比达9.4％[9]，且有多个产品上市[19]，是相关研究和应用的主要选择之一。相较而言，非病毒载体则仍处于早期探索阶段[8]。

二、HB基因治疗Ⅲ期临床试验概览

当前HB基因治疗已完成Ⅲ期临床试验包括Etranacogene Dezaparvovec、Fidanacogene Elaparvovec和BBM-H901，其中前两款产品已在部分国家获批上市。

（一）Etranacogene Dezaparvovec

Etranacogene Dezaparvovec由AAV5-FⅨ（Padua）构成[22]。HOPE-B Ⅲ期临床试验以2×10^13^ vg/kg剂量行单次静脉输注Etranacogene Dezaparvovec，54例受试者随访18个月后平均稳态FⅨ水平39％，治疗后年化出血率（ABR）从治疗前4.19下降到1.51，零出血患者比例由26％增加至63％，年化输注FⅨ平均减少248 825 IU，96％的受试者停止预防性输注FⅨ。主要不良事件为肝酶升高（20％），未发生严重的治疗相关不良事件（TRAE），也未出现FⅨ抑制物[22]。试验后延长随访至3年，平均FⅨ活性可保持在38.6％左右，ABR为1.52，关节出血发生率降低80％，无关节出血率达88.5％，血友病关节健康总体评分从21.7降至18.2，关节获益表现出时间依赖性的递增[23]。

（二）Fidanacogene Elaparvovec

Fidanacogene Elaparvovec由AAV（Spark100）-FⅨ（Padua）构成[24]。BENEGENE-2 Ⅲ期临床试验以5×10^11^ vg/kg剂量行单次静脉输注，45例受试者随访15个月后平均稳态FⅨ水平26.9％，治疗后ABR由治疗前的4.42降至1.28，零出血比例由29％提升至64％，平均年化输注FⅨ减少了2 929.17 IU/kg。有13％的患者治疗后仍需要预防性输注FⅨ。试验期间62％的患者因肝酶升高和/或FⅨ活性降低接受了糖皮质激素治疗，未发生严重治疗相关AE或血栓形成[24]。纳入了Fidanacogene Elaparvovec所有受试者（75％来自BENEGENE-2试验，中位随访2.8年）的长期随访结果显示，最常见的不良事件是肝酶升高（20％），严重不良事件为贫血（3％），所有受试者在输注载体1年后均未再接受糖皮质激素治疗[25]。

（三）BBM-H901

BBM-H901是亚洲首个进入临床试验的HB基因治疗药物，由AAV843-FⅨ（Padua）构成[26]。BBM-H901的Ⅲ期临床试验以5×10^12^ vg/kg剂量单次静脉输注，3 d后26例受试者平均FⅨ水平即升至49.70 IU/dl，52周后平均稳态FⅨ水平55.08 IU/dl，治疗后ABR平均值从治疗前的5.0（与监管机构商定，以国内预防治疗人群的ABR均值为受试者基线ABR均值）降至0.6，FⅨ输注平均数由58.2次降至2.9次，81％的患者停止替代治疗，出血关节平均数从1.1降至0，未出现3级及以上TRAE。试验期间最常见的1～2级TRAE包括丙氨酸转氨酶（ALT）升高（26.9％）、天冬氨酸转氨酶升高（7.7％）和纤维蛋白原降低（11.5％）[27]。此外，在先期进行的一项由中国医学科学院血液病医院进行的研究者发起的研究中，受试者已随访超过3年，平均FⅨ水平维持在37％以上[26]。特别是1例患者在接受BBM-H901治疗近16个月后，于膝关节置换术的围术期未输注FⅨ，成为全球首例在无替代治疗下完成大手术的HB患者，显示了基因治疗后患者体内FⅨ（Padua）活性可高于50％（常用于大手术FⅨ活性下限），能够满足高要求手术的止血需要[28]。

除上述三种药物的Ⅲ期试验之外，还有FLT180a、BAX 335和CHOP等其他多个HB基因治疗产品曾进入临床试验，在AAV血清型、FⅨ基因型和剂量选择上均存在不同程度的差异，早期结果显示其FⅨ表达活性和持续时长也存在差异[29]。

三、讨论和展望

（一）肝脏免疫反应与免疫抑制剂的使用

HB基因治疗的最终目标在于寻求FⅨ能够持久稳定表达，进而使患者达到功能性治愈。已有小规模研究观察到基因治疗后FⅨ活性水平可持续8年以上且没有下降趋势，模型预测也显示Etranacogene Dezaparvovec治疗后FⅨ活性水平可提升长达25.5 年，80％以上的HB患者在此期间不需要预防性使用因子替代治疗[30]。然而仍需要更大规模、更长期的随访证实基因治疗的长期有效性。国际性基因疗法登记系统已因此上线，用于收集所有基因治疗的载体细节、安全性、有效性、生活质量和疾病负担等[31]。

基因治疗目的基因的表达受到载体剂量、载体结构或血清型、特异性启动子、转基因效率和宿主自身的多重影响[7]，但对其具体过程了解有限。作为HB基因治疗的靶细胞，肝细胞的病生理状态亦会直接影响转基因的长期疗效[19]，而特异性免疫和非特异性免疫均可导致肝细胞的免疫损伤[32]。现有临床试验普遍观察到部分患者接受基于AAV的基因治疗后可出现无症状肝转氨酶升高等表现，这可能与CD8^+^ T细胞对AAV衣壳中特定序列的免疫反应有关，由此不仅可能损伤或破坏肝细胞进而造成凝血因子生成不足或表达时间缩短，还可能与凝血因子蛋白未折叠相关[19]–[20],[32]，最终导致一些患者转基因表达的部分或全部丢失[33]。这提示针对肝细胞的特异性免疫应答强度及持续时间对FⅨ的长期表达具有重要影响。非特异性免疫应答系统的激活也可损伤肝细胞，可能途径包括AAV载体表达框富含CpG寡核苷酸序列可能经Toll样受体诱发非特异性免疫，以及大剂量（5×10^13^～2×10^14^ vg/kg）的AAV可激活补体系统等[32],[34]–[35]。

对于基因治疗通过特异性免疫应答途径诱发的肝细胞损伤，早期的一项试验表明激素短期治疗可降低肝转氨酶并维持目的基因的表达[15]，此后糖皮质激素得到广泛使用，并在多个试验中验证了激素对FⅨ表达的维持作用[32]。值得注意的是，HOPE-B和BENEGENE-2试验均在基因输注后肝转氨酶升高（ALT高于正常值上限或为基线值的2倍）和（或）FⅨ活性下降时反应性启动糖皮质激素治疗，而BBM-H901的临床试验则首次表明，HB患者基因治疗前预防性糖皮质激素治疗可有效抑制细胞对AAV的免疫应答，绝大部分患者的肝转氨酶不超过正常值上限，确保了基因治疗的有效性[27],[36]。GENEr8-3试验则在输注载体的同时预防性启动激素治疗，随访1年的结果和GENEr8-1试验比较，激素降低了血友病A患者ALT高于正常值上限的发生率，但没有降低高于正常值上限的ALT平均峰值，而且86.4％的患者出现了糖皮质激素相关不良事件[37]。这两项研究尽管不能完全确定预防性使用糖皮质激素可以最小化基因治疗导致的肝细胞损伤、更持久地维持FⅨ表达，但很可能有助于降低肝损伤的发生率，未来有望为某些基因治疗后肝损伤潜在风险升高的HB患者提供可选的管理手段。

除了糖皮质激素外，硫唑嘌呤、霉酚酸酯和他克莫司等药物有望用于抑制基因治疗后的免疫应答与炎症反应，其药理机制也各不相同。对于血友病基因治疗后糖皮质激素与其他免疫抑制剂的选择、剂量、疗程、序贯或联合等用法问题目前尚未达成共识。

免疫抑制疗法并非对所有接受基因治疗的患者都有效。已知AAV衣壳引发的肝脏免疫毒性具有剂量依赖性特征[20],[34]，大剂量时可激活非特异性免疫应答系统，由此诱发的肝细胞损伤不能通过糖皮质激素治疗缓解，FⅨ表达最终丢失[32],[34]–[35]。然而，即使去除载体激活非特异性免疫的特定序列、限定给药剂量并予糖皮质激素，仍出现FⅨ表达显著降低的案例，原因未知[36],[38]。基因治疗后在肝酶升高时行肝活检以分析原位免疫反应和基因表达的情况，可能有助于进一步揭示目的基因表达丧失的机制。

（二）诱发恶性肿瘤

2001年报告在乳鼠中发现AAV对肝细胞具有插入诱变毒性[39]，2015年报告在人类肝细胞癌组织中发现野生型AAV2的克隆整合[40]，这引起了AAV2是否为人体细胞致癌因素的争议。此后大多数研究发现AAV没有增加成年小鼠肝细胞癌的风险，并且AAV整合进入犬类DNA的现象罕见，而对出现DNA整合的犬只观察10年也未发现细胞癌变[34],[40]。从机制上分析，乳鼠肝细胞癌与AAV插入Rian基因座并导致临近的基因表达失调有关，但人类DNA并不存在Rian基因座[39]。更重要的是，目前作为载体的AAV已删除Rep蛋白，这使AAV基因组整合宿主DNA的概率降至极低[41]。虽然已报告2例血友病患者在接受AAV基因治疗后分别出现了肝癌和腮腺癌，但病理检查和基因分析表明癌变与AAV无关[32],[42]，因此临床试验也已初步证实AAV基因疗法不会诱发恶性肿瘤。另外，目前正在探索非靶向肝细胞基因疗法，可能为某些肝细胞癌潜在高风险的HB患者提供替代选择[43]。

（三）生殖毒性

既往在某些男性不育症的患者精液中观察到野生型AAV基因的存在，一些流产的发生亦可能与胚胎和宫颈上皮细胞感染野生型AAV有关[41]，这可能会引起对AAV生殖毒性的担忧，但基因重组后的AAV相关研究结果并不支持这一点。早期实验表明，基因重组AAV或AAV-FⅨ输注后会短暂出现于受试动物精液中，但不会转导进入精子细胞，子代也没有基因整合的现象[44]–[46]。近年来对小鼠精原干细胞或早期胚胎细胞的基因转导实验观察到，在局部注射或体外培养条件下，某些血清型AAV可进入细胞，但不会整合到细胞DNA或子代基因组，且在胞内存在的时间短暂[47]–[49]。已有的临床试验显示，尽管接受了不同药物、不同剂量的基因治疗，患者均可在不同时间内清除精液中的AAV基因[22],[26]。因此，目前的研究提示AAV基因治疗意外导致患者生殖毒性的风险极低，但更可靠的结论依然有赖于更大规模的长期随访。

四、结论

随着相关基因治疗药物先后获批上市，HB的临床实践正在进入基因治疗时代。由于FⅨ有望得到长期而稳定的表达，治愈已成为中重度HB的潜在临床治疗目标，患者完全回归正常生活将成为可能。尽管对基于AAV的基因疗法仍然存在众多尚未完全了解或确证的课题，但随着对相关患者临床随访的延续和更多试验及临床应用的开展，未来有望由此获得更坚实的研究成果，这一新型疗法也有望更好地造福HB患者。
